# Supplementary material for: Modeling glioblastoma heterogeneity as a dynamic network of cell states
Source: Mol Syst Biol. 2021 Sep 16;17(9):e10105. doi: 10.15252/msb.202010105 (PMC8444284; doi:10.15252/msb.202010105)
Supplement: Supplementary file 6 — Source Data for Figure 5 [file MSB-17-e10105-s004.zip › Figure5A_sourcedata/GSEA_3017/nosigdb_stateE.GseaPreranked.1622795031093/RICHARDS_NEFTEL_CELL_2019_MES1.html]

Details for gene set RICHARDS\_NEFTEL\_CELL\_2019\_MES1[GSEA]

|  || Dataset | state63017 |
| Phenotype | NoPhenotypeAvailable |
| Upregulated in class | na\_pos |
| GeneSet | RICHARDS\_NEFTEL\_CELL\_2019\_MES1 |
| Enrichment Score (ES) | 0.79109246 |
| Normalized Enrichment Score (NES) | 3.3639019 |
| Nominal p-value | 0.0 |
| FDR q-value | 0.0 |
| FWER p-Value | 0.0 |
Table: GSEA Results Summary

  

Fig 1: Enrichment plot: RICHARDS\_NEFTEL\_CELL\_2019\_MES1      
 Profile of the Running ES Score & Positions of GeneSet Members on the Rank Ordered List

  

| PROBE | GENE SYMBOL | GENE\_TITLE | RANK IN GENE LIST | RANK METRIC SCORE | RUNNING ES | CORE ENRICHMENT || 1 | ANXA1 |  |  | 12 | 0.826 | 0.0808 | Yes |
| 2 | CD44 |  |  | 14 | 0.806 | 0.1712 | Yes |
| 3 | RCAN1 |  |  | 17 | 0.750 | 0.2541 | Yes |
| 4 | ANXA2 |  |  | 18 | 0.733 | 0.3372 | Yes |
| 5 | LGALS3 |  |  | 25 | 0.697 | 0.4099 | Yes |
| 6 | IFITM3 |  |  | 26 | 0.682 | 0.4873 | Yes |
| 7 | TAGLN2 |  |  | 32 | 0.621 | 0.5524 | Yes |
| 8 | GJA1 |  |  | 33 | 0.618 | 0.6226 | Yes |
| 9 | EMP1 |  |  | 40 | 0.584 | 0.6824 | Yes |
| 10 | S100A10 |  |  | 51 | 0.527 | 0.7315 | Yes |
| 11 | SERPINE1 |  |  | 52 | 0.526 | 0.7911 | Yes |
| 12 | LGALS1 |  |  | 113 | 0.368 | 0.7684 | No |
| 13 | A2M |  |  | 184 | 0.309 | 0.7282 | No |
| 14 | SPP1 |  |  | 261 | 0.257 | 0.6758 | No |
| 15 | WWTR1 |  |  | 263 | 0.256 | 0.7038 | No |
| 16 | TUBA1C |  |  | 282 | -0.253 | 0.7132 | No |
Table: GSEA details [plain text format]

  

Fig 2: RICHARDS\_NEFTEL\_CELL\_2019\_MES1: Random ES distribution      
 Gene set null distribution of ES for **RICHARDS\_NEFTEL\_CELL\_2019\_MES1**
